# High Prevalence of Asymptomatic Intracranial Atherosclerosis in Elder Women With Tubal Ligation: Result From a Community-Based Study in Shandong, China

**DOI:** 10.3389/fcvm.2022.830068

**Published:** 2022-03-02

**Authors:** Xiaotong Ma, Shaowei Sang, Yuanyuan Zhao, Xiang Wang, Xiaokang Ji, Sai Shao, Guangbin Wang, Fuzhong Xue, Yifeng Du, Ming Lv, Qinjian Sun

**Affiliations:** ^1^Department of Neurology, Shandong Provincial Hospital Affiliated to Shandong First Medical University, Jinan, China; ^2^Department of Clinical Epidemiology, Qilu Hospital, Cheeloo College of Medicine, Shandong University, Jinan, China; ^3^Department of Biostatistics, School of Public Health, Shandong University, Jinan, China; ^4^Department of Radiology, Shandong Provincial Hospital Affiliated to Shandong First Medical University, Jinan, China

**Keywords:** asymptomatic intracranial atherosclerosis, menstrual history, reproductive history, tubal ligation, population surveillance

## Abstract

**Background:**

In addition to traditional cardiovascular risk factors, gender-specific factors may also contribute to intracranial atherosclerosis. This study aimed to comprehensively investigate the association between asymptomatic intracranial atherosclerosis (aICAS) and menstrual or reproductive history (MRH), namely, menstruation, pregnancy, childbirth, menopause, and contraception.

**Methods:**

Participants in this study were selected from the Kongcun town aICAS study. MRH was collected through structured case report forms, in which menarche age, menstrual regularity, dysmenorrhea, number of pregnancies, number of childbirths, age of first pregnancy, breastfeeding, menopause, menopause age, and contraceptive methods were all involved. All characteristics were compared by chi-squared and nonparametric tests as applicable. Logistic regression model and sensitivity analysis were used to analyze the association between aICAS and MRH.

**Results:**

A total of 1,052 female participants were involved in this study, of which 5.7% had moderate to severe aICAS. Tubal ligation was significantly associated with aICAS in univariate analysis [crude odds ratio (OR), 2.85; 95% CI, 1.22–6.62; *P* = 0.015]. This association was still significant among female participants over 60 years old after multivariate adjustment (adjusted OR, 4.36; 95% CI, 1.55–12.24; *P* = 0.005). Sensitivity analysis showed a similar result (adjusted OR, 3.76; 95% CI, 1.24–11.41; *P* = 0.020). Menopause lost significant association with aICAS after multivariate adjustment (adjusted OR, 1.68; 95% CI, 0.66–4.24; *P* = 0.275). No other MRH factors were found to be associated with aICAS.

**Conclusion:**

Tubal ligation may be associated with a higher prevalence of aICAS in Chinese elderly women. This provides a new perspective to study the epidemiological characteristics of ICAS.

## Introduction

Intracranial atherosclerosis (ICAS) is an important cause of stroke and has attracted more and more attention, especially in high-incidence areas such as China (1, 2). ICAS can remain asymptomatic for many years until transient ischemic attack (TIA) or stroke occured (3). Early detection and prevention of ICAS in the asymptomatic stage are of great importance.

A number of studies had been devoted to finding the epidemiological risk factors for the development of ICAS. It was generally believed that asymptomatic ICAS (aICAS) can be largely attributed to traditional cardiovascular risk factors, such as hypertension, diabetes, and dyslipidemia (4–7). However, increasing evidence suggested that traditional risk factors cannot fully explain the epidemiological characteristics of aICAS (2).

Elder women were more likely to develop aICAS than men (8). Recent studies had shown that gender-specific factors might also play a specific role in the development of ICAS (9). Menstrual or reproductive history (MRH) was an important gender-related clinical characteristic of women, which included data on menstruation, menopause, pregnancy, childbirth, and sterilization. It was rich in information and relatively easy to collect in clinical. Several previous studies had indicated that MRH was closely related to atherosclerotic diseases of peripheral arteries, coronary arteries, and carotid arteries, which could assist risk prediction (10–12). Only the Asymptomatic Polyvascular Abnormalities Community study (APAC) had tried to find the association between postmenopausal time and newly discovered aICAS with a negative result (8). Few other studies have analyzed the correlation between MRH and aICAS before.

Therefore, we investigated the detailed information of MRH among female residents in Kongcun Town, Shandong Province, China, and explored its relationship with aICAS, trying to find the potential risk factors of aICAS from a new perspective to help early identification of aICAS in the future.

## Methods

### Study Population and Study Design

This was a cross-sectional study of a single-center cohort of healthy residents. Participants in this study were selected from female residents in the Kongcun Town aICAS (KT-aICAS) study, which had been described in detail before (13). Briefly, there were 1,064 female participants from Kongcun Town who were over 40 years old and free of TIA and stroke. For all participants with written informed consent, MRH and epidemiological characteristics were retrospectively collected through face-to-face interviews by case report forms. Serological examination, carotid ultrasound, and transcranial Doppler (TCD) were also conducted, and magnetic resonance angiography (MRA) was further performed on participants with aICAS detected by TCD.

This study was approved by the Human Experiment Ethics Standards Committee of Shandong Provincial Hospital Affiliated with Shandong First Medical University and was carried out in accordance with the Declaration of Helsinki (14).

### Detection of Cerebral Vascular Atherosclerosis

Asymptomatic ICAS was detected in two steps by TCD and MRA, respectively (15, 16). TCD was carried out for all participants by two physicians with a portable machine (VIASYS Companion III). Bilateral middle cerebral artery, internal carotid artery, anterior cerebral artery, posterior cerebral artery, vertebral artery, and basilar artery were examined with a 2-MHz probe *via* temporal, occipital, and eye windows. The criteria for judging the degree of stenosis detected by TCD were described in detail by Wang et al. (13). Moderate to severe stenosis of more than 50% was defined as positive results.

For participants who were defined as positive by TCD, MRA was further performed on a 3.0 T Philips magnetic resonance scanner. The degree of intracranial atherosclerosis stenosis on MRA was jointly interpreted by a neurologist and a radiologist based on the degree of signal reduction (13). Asymptomatic ICAS was eventually determined as stenosis over 50% on both the TCD and MRA.

For all the participants, a carotid artery ultrasound was performed on the Siemens ACUSON P500 system by two experienced sonographers using 7-MHz linear sensors. Common carotid artery, internal carotid artery, and external carotid artery were scanned longitudinally and transversally with participants in the supine position with head straight and flat. Atherosclerosis plaque was defined as focal intima-media thickness ≥ 1.5 mm (13). Extracranial atherosclerosis (ECAS) was defined as any degree of stenosis in one or more arteries of the common carotid artery, internal carotid artery, and external carotid artery according to the established carotid ultrasonography criteria (17).

### Case Report Form

Hypertension was defined as blood pressure ≥ 140/90 mm Hg, taking antihypertensive drugs, or previously diagnosed hypertension. Diabetes was defined as plasma glucose ≥ 7.0 mmol/l, taking hypoglycemic drugs, or previously diagnosed diabetes. Dyslipidemia was defined as total cholesterol (TC) ≥ 6.20 mmol/l, triglyceride (TG) ≥ 1.80 mmol/l, high-density lipoprotein (HDL) <1.11 mmol/l, low-density lipoprotein (LDL) ≥ 3.36 mmol/l, use of cholesterol-lowering medication, or self-reported hyperlipidemia. Body mass index (BMI) was calculated as the ratio of weight and square of height (m). The waist circumference was measured from the midpoint of the lumbar ridge and the lower edge of the ribs. The hip circumference was measured from the greater trochanter of the femur. Both waist and hip circumferences were measured in a standing position when fasting. Waist-to-hip ratio (WHR) was calculated as the ratio of waist circumference to hip circumference. Smoking was defined as at least one cigarette per day in the past 6 months. Drinking was defined as alcohol intake at least once a week within the past 6 months. Plasma glucose, LDL, HDL, TC, TG, hypersensitive C-reactive protein (hs-CRP), and homocysteine (HCY) were tested in the laboratory of Provincial Hospital Affiliated to Shandong First Medical University.

For the menstruation history, we collected data on menarche age, menstrual regularity, menstrual interval, menstrual duration, dysmenorrhea, menopause, etc. Menarche age was the age at first menstruation. Menstrual regularity was primarily determined by the participants' self-reports, supplemented by the definition of regular menstruation that occurred within 7 days before or after the expected time. For participants with regular menstruation, the menstrual interval and duration were recorded as the average of the longest and shortest intervals and durations, respectively. Dysmenorrhea was divided into never, sometimes, and always according to its frequency. Menopause was primarily determined by the participants' self-reports, supplemented by the definition of the absence of menstruation for more than 1 year. Unnatural menopause referred to menopause caused by uterine or ovarian surgery, drugs, or other special reasons. For women who had been menopausal, the age of menopause, and the time after menopause was also recorded.

For the reproductive history, we collected data on the number of pregnancies, the age at first pregnancy, adverse pregnancy outcomes, the number of childbirths, breastfeeding, and contraceptive methods. The number of pregnancies was counted including miscarriage and induction of labor. The number of deliveries was counted including stillbirth. Adverse pregnancy outcomes referred to unexpected miscarriage and stillbirth. The feeding method was classified as breastfeeding and nonbreastfeeding. Contraceptive methods were generally divided into oral contraceptives, condom contraception, intrauterine device contraception, and tubal ligation. Contraceptive contraception specifically referred to the medical history of taking oral contraceptives regularly. Occasional use of emergency contraceptives was not included. The starting age of contraceptives and the duration of drug taking were also recorded. There was no detailed distinction between the types of intrauterine devices. According to the medical records of the local health center, the Irving method was used for the local female residents over 40 years old for tubal ligation.

### Statistical Analysis

Except for those who refuse to disclose their menstrual or reproductive history, all data were retained to the greatest extent after data cleaning and logical inspection. Missing values were filled manually with reference to the mode and the median. Kolmogorov–Smirnov test was used to assess the normality of data. Continuous variables with skewed distribution were represented by the median with Q1–Q3. Categorical variables were represented by frequency and percentage. Variables were compared using the Mann–Whitney *U* test or chi-squared test as applicable. The logistic regression model was used to estimate the adjusted association between MRH and aICAS with an odds ratio (OR) and 95% CI. The change-in-estimate method was used to get confounding factors included in the multivariate adjustment (18). We also performed a sensitivity analysis deleting all missing values. Two-tail test with a significant *P* < 0.05 was used for analysis. All statistical analyses were performed by IBM SPSS Statistics V22.0 for Windows (IBM Corporation, Released 2013, Armonk, New York, USA).

## Results

### Profile of the Study Population

**Figure 1:**
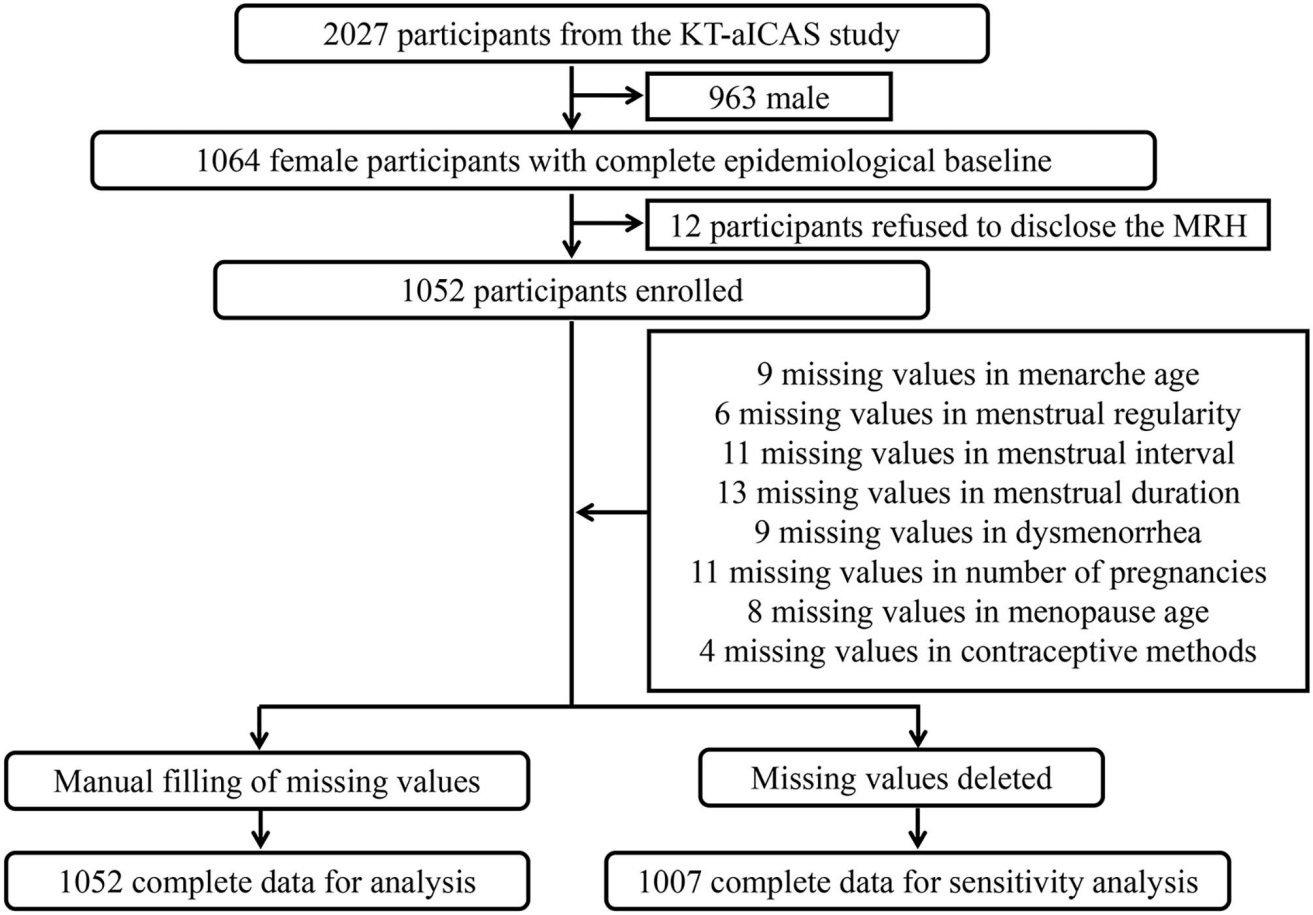
Flowchart.

Among the 1,064 female participants from the KT-aICAS study, 12 rejected the survey of the menstrual or reproductive history, and a total of 1,052 participants were eventually included in this study (Figure 1). As shown in Table 1, 1,052 participants ranged from 40 to 90 years old, with a median age of 58 years old. It was found that 60 out of the 1,052 participants had moderate to severe aICAS through TCD and MRA, in which 33 out of 447 were over 60 years old. Among the 1,052 participants, 23 had varying degrees of asymptomatic ECAS detected by carotid ultrasound. The prevalence of hypertension, diabetes, and dyslipidemia among study participants were 58.7, 18.2, and 45.4%, respectively. The median age at menarche of the participants was 16 years old, of which 86.2% had regular menstrual cycles and 54.8% had dysmenorrhea. There were 1,047 participants with a history of pregnancy, of which 490 had three or more pregnancies and 335 had three or more childbirths. The median age of their first pregnancy was 24 years old. A total of 816 participants had menopause, of which 4.3% was unnatural menopause. The median age of menopause was 50 years old. Forty-three percentage of the participants used intrauterine devices for contraception, 4.8% underwent tubal ligation, and 2.2 and 0.6% of participants chose condoms and contraceptives, respectively.

**Table 1:** The epidemiological baseline and MRH characteristics of female participants.

**Characteristics**	**All participants**	**with aICAS**	**without aICAS**	***P-*value**
	**(*n* = 1,052)**	**(*n* = 60)**	**(*n* = 992)**	
Age, years	58.0 (51.0–66.0)	63.0 (52.5–70.0)	58.0 (51.0–66.0)	0.025^*^
>60 years old, (%)	447 (42.5)	33 (55.0)	414 (41.7)	0.059
≤ 60 years old, (%)	605 (57.5)	27 (45.0)	578 (58.3)	
Hypertension, (%)	617 (58.7)	49 (81.7)	568 (57.3)	<0.001^***^
Diabetes, (%)	191 (18.2)	19 (31.7)	172 (17.3)	0.009^**^
Dyslipidemia, (%)	478 (45.4)	32 (53.3)	446 (45.0)	0.230
LDL, mmol/l	3.06 (2.64–3.53)	3.23 (2.86–3.84)	3.04 (2.63–3.49)	0.002^**^
HDL, mmol/l	1.58 (1.38–1.83)	1.49 (1.32–1.64)	1.59 (1.38–1.83)	0.010^*^
TG, mmol/l	1.22 (0.89–1.72)	1.58 (1.10–2.26)	1.20 (0.89–1.69)	0.001^**^
TC, mmol/l	5.40 (4.78–6.12)	5.53 (5.03–6.45)	5.39 (4.77–6.10)	0.083
BMI, kg/m^2^	25.15 (23.10–27.67)	26.47 (24.09–28.86)	25.08 (22.97–27.57)	0.005^**^
WHR	0.90 (0.85–0.95)	0.94 (0.89–0.98)	0.90 (0.85–0.95)	0.006^**^
Hs-CRP, mg/l	0.79 (0.32–1.74)	0.98 (0.58–2.17)	0.78 (0.30–1.71)	0.016^*^
HCY, μmol/l	12.77 (10.94–15.38)	12.99 (10.18–15.01)	12.77 (10.95–15.45)	0.414
Smoking, (%)	12 (1.1)	0	12 (1.2)	-
Drinking, (%)	43 (4.1)	3 (5.0)	40 (4.0)	0.732
ECAS, (%)	23 (2.2)	6 (10.0)	17 (1.7)	0.001^**^
Hypolipidemic drug, (%)	16 (1.5%)	0	16 (1.6%)	-
Menarche age, years	16.0 (15.0–18.0)	16.0 (15.0–18.0)	16.0 (15.0–18.0)	0.330
Regular menstruation, (%)	907 (86.2)	49 (81.7)	858 (86.5)	0.332
Menstrual interval, days	29.0 (27.5–30.0)	29.5 (28.25–30.0)	29.0 (27.5–30.0)	0.096
Menstrual duration, days	4.5 (3.5–5.5)	4.5 (3.5–5.5)	4.5 (3.5–5.5)	0.796
Dysmenorrhea, (%)				0.190
Never	475 (45.2)	21 (35.0)	454 (45.8)	
Sometimes	277 (26.3)	21 (35.0)	256 (25.8)	
Always	300 (28.5)	18 (30.0)	282 (28.4)	
Age of first pregnancy, years	24.0 (22.0–26.0)	24.0 (22.0–26.0)	24.0 (22.0–26.0)	0.928
Number of pregnancies	2 (2–3)	3 (2–4)	2 (2–3)	0.504
<3 times, (%)	563 (53.5)	29 (48.3)	534 (53.8)	0.426
≥3 times, (%)	489 (46.5)	31 (51.7)	458 (46.2)	
Adverse pregnancy outcomes, (%)	289 (27.5)	14 (23.3)	275 (27.7)	0.552
Number of deliveries	2 (2–3)	2 (2–3)	2 (2–3)	0.302
<3 times, (%)	717 (68.2)	37 (61.7)	680 (68.5)	0.318
≥3 times, (%)	335 (31.8)	23 (38.3)	312 (31.5)	
Breastfeeding, (%)	993 (94.4)	53 (88.3)	940 (94.8)	0.073
Menopause, (%)	816 (77.6)	53 (88.3)	763 (76.9)	0.039^*^
Unnatural menopause, (%)	35 (3.3)	4 (6.7)	31 (3.1)	0.280
Menopausal age, years	50.0 (47.0–51.0)	49.0 (47.0–51.5)	50.0 (47.0–51.0)	0.850
Postmenopausal time, years	12.0 (5.0–20.0)	15.0 (4.5–22.0)	12.0 (5.0–20.0)	0.496
Oral contraceptives, (%)	6 (0.6)	0	6 (0.6)	-
Condom, (%)	23 (2.2)	1 (1.7)	22 (2.2)	-
Intrauterine device, (%)	452 (43.0)	24 (40.0)	428 (43.1)	0.688
Tubal ligation, (%)	51 (4.8)	7 (11.7)	44 (4.4)	0.022^*^

*Continuous variables were represented as median and Q1–Q3. Categorical variables were represented as frequency and percentage. MRH, menstrual or reproductive history; aICAS, asymptomatic intracranial atherosclerosis; LDL, low-density lipoprotein; BMI, body mass index; WHR, waist-to-hip ratio; Hs-CRP, high sensitivity C-reactive protein; HCY, homocysteine*.

* *represent for p-value < 0.05;*

** *represent for p-value < 0.01;*

*** *represent for p-value < 0.001*.

### Asymptomatic ICAS and Its Related Factors

Women with aICAS tended to be older, with higher levels of LDL, TG, BMI, WHR, and Hs-CRP, and lower levels of HDL. Compared with women without aICAS, women with aICAS also have a higher burden of hypertension, diabetes, and asymptomatic extracranial atherosclerosis (Table 1).

In the univariate analysis of MRH and aICAS, menopausal women and women with tubal ligation were more likely to develop aICAS (*P* = 0.039 and 0.022, respectively). Other factors of menstrual or reproductive history, such as menarche age, menstrual regularity, dysmenorrhea, number of pregnancies, number of childbirths, and use of other contraceptive methods were not significantly associated with aICAS (Table 1).

### Association Between Tubal Ligation and aICAS

Menopause lost its relevance to aICAS after taking age into account (Model 1: adjusted OR, 1.68; 95% CI, 0.66–4.24). While tubal ligation was still significantly associated with a higher prevalence of aICAS after adjusting for age (Model 1: adjusted OR, 2.80; 95% CI, 1.20–6.54) (Table 2).

**Table 2:** Association of tubal ligation with asymptomatic intracranial arterial stenosis in the logistic regression model.

	**Model 1**		**Model 2**		**Model 3**	
	**Adjusted OR**	***P-*value**	**Adjusted OR**	***P-*value**	**Adjusted OR**	***P-*value**
	**(95% CI)**		**(95% CI)**		**(95% CI)**	
Menopause	1.68 (0.66–4.24)	0.275	-	-	-	-
Tubal ligation	2.80 (1.20–6.54)	0.017	2.11 (0.87–5.11)	0.097	1.97 (0.82–4.72)	0.130
>60 years old	5.27 (1.92–14.52)	0.001	4.61 (1.52–13.97)	0.007	4.36 (1.55–12.24)	0.005
≤ 60 years old	0.77 (0.10–5.86)	0.797	0.28 (0.03–2.97)	0.292	0.38 (0.04–3.26)	0.374
**Sensitivity analysis**						
**Tubal ligation**
>60 years old	4.81 (1.61–14.37)	0.005	3.38 (1.03–11.14)	0.045	3.76 (1.24–11.41)	0.020

*Model 1 was adjusted for age. Model 2 was adjusted for age, hypertension, diabetes, LDL, HDL, BMI, WHR, ECAS, hs-CRP, and menopause. Model 3 was adjusted for hypertension, HDL, and LDL according to the change-in-estimate method. OR, odds ratio; CI, confidence interval; LDL, low-density lipoprotein; HDL, high-density lipoprotein; BMI, body mass index; WHR, waist-to-hip ratio; ECAS, extracranial atherosclerosis; Hs-CRP, high sensitivity C-reactive protein*.

We performed a full adjustment for tubal ligation in Model 2 with age, hypertension, diabetes, LDL, HDL, TG, BMI, WHR, Hs-CRP, ECAS, and menopause. According to the change-in-estimate method, hypertension, HDL, and LDL were selected as confounding factors for adjustment in Model 3. Tubal ligation was still significantly associated with aICAS in the age group over 60 years old after multivariate adjustment (Model 2: adjusted OR, 4.61; 95% CI, 1.52–13.97. Model 3: adjusted OR, 4.36; 95% CI, 1.55–12.24). However, tubal ligation lost its significant association with aICAS among participants of all ages (Model 2: adjusted OR, 2.11; 95% CI, 0.87–5.11 and Model 3: adjusted OR, 1.97; 95% CI, 0.82–4.72) (Table 2).

Sensitivity analysis was performed with all missing values deleted. A total of 1,007 participants were included in the sensitivity analysis, of which 56 had aICAS and 47 had undergone tubal ligation. Consistent with previous results, there was still a significant association between tubal ligation and aICAS among women over 60 years old after correcting for potential confounding factors (Model 2: adjusted OR, 3.38; 95% CI, 1.03–11.14 and Model 3: adjusted OR, 3.76; 95% CI, 1.24–11.41) (Table 2).

### Characteristics of Women With Tubal Ligation

To better understand the characteristics of the study population, we also compared the MRH and epidemiological baseline of women with or without tubal ligation. Compared with unligated women, ligated women had significantly more pregnancies (*P* = 0.013) and childbirths (*P* = 0.011). At the same time, women with tubal ligation tend to have a higher proportion of hypertension (*P* = 0.003) and higher levels of LDL (*P* = 0.035) (Table 3).

**Table 3:** The epidemiological characteristics and MRH between women with or without tubal ligation.

**Characteristics**	**Participants with tubal ligation**	**Participants without tubal ligation**	***P*-value**
	**(*n* = 51)**	**(*n* = 1,001)**	
Age, years	58.0 (52.0–67.0)	58.0 (51.0–66.0)	0.393
>60 years old, (%)	23 (45.1)	424 (42.4)	0.772
≤ 60 years old, (%)	28 (54.9)	577 (57.6)	
Hypertension, (%)	40 (78.4)	577 (57.6)	0.003^*^
Diabetes, (%)	9 (17.6)	182 (18.2)	1.000
Dyslipidemia, (%)	27 (52.9)	451 (45.1)	0.313
TG, mmol/l	1.40 (1.03–1.87)	1.21 (0.89–1.72)	0.118
TC, mmol/l	5.68 (4.92–6.35)	5.39 (4.78–6.12)	0.160
LDL, mmol/l	3.23 (2.83–3.75)	3.04 (2.63–3.51)	0.035^*^
HDL, mmol/l	1.51 (1.31–1.74)	1.59 (1.38–1.83)	0.084
BMI, kg/m^2^	25.54 (23.46–28.32)	25.15 (23.01–27.59)	0.417
WHR	0.91 (0.84–1.00)	0.90 (0.85–0.95)	0.562
Hs-CRP, mg/l	0.84 (0.37–1.70)	0.79 (0.31–1.75)	0.705
HCY, μmol/l	13.17 (10.94–16.85)	12.75 (10.94–15.33)	0.290
Smoking, (%)	0	12 (1.2)	-
Drinking, (%)	5 (9.8)	38 (3.8)	0.052
ECAS, (%)	1 (2.0)	22 (2.2)	-
aICAS, (%)	7 (13.7)	53 (5.3)	0.022^*^
Hypolipidemic drug, (%)	0	16 (1.6)	-
Menarche age, years	17.0 (15.0–18.0)	16.0 (15.0–18.0)	0.681
Regular menstruation, (%)	41 (80.4)	866 (86.5)	0.213
Menstrual interval, days	29.5 (28.5–30.0)	29.0 (27.5–30.0)	0.237
Menstrual duration, days	4.0 (3.5–5.5)	4.5 (3.5–5.5)	0.694
Dysmenorrhea, (%)			0.327
Never	27 (52.9)	448 (44.8)	
Sometimes	14 (27.5)	263 (26.3)	
Always	10 (19.6)	290 (29.0)	
Age of first pregnancy, years	24.0 (22.0–25.0)	24.0 (22.0–26.0)	0.309
Number of pregnancies	3 (2–4)	2 (2–3)	0.013^*^
<3 times, (%)	23 (45.1)	540 (53.9)	0.250
≥3 times, (%)	28 (54.9)	461 (46.1)	
Adverse pregnancy outcomes, (%)	7 (13.7)	81 (8.1)	0.189
Number of deliveries	2 (2–4)	2 (2–3)	0.011^*^
<3 times, (%)	29 (56.9)	688 (68.7)	0.090
≥3 times, (%)	22 (43.1)	313 (31.3)	
Breastfeeding, (%)	47 (92.2)	946 (94.5)	0.524
Menopause, (%)	44 (86.3)	772 (77.1)	0.167
Unnatural menopause, (%)	3 (5.9)	32 (4.2)	0.426
Menopausal age, years	49.0 (47.0–50.0)	50.0 (47.0–51.0)	0.108
Postmenopausal time, years	12.0 (5.0–19.0)	12.0 (5.0–20.0)	0.825
Oral contraceptives	0	6 (0.6)	-
Condom	0	23 (2.3)	0.623
Intrauterine device	17 (33.3)	435 (43.5)	0.192

*Continuous variables were represented as median and Q1–Q3. Categorical variables were represented as frequency and percentage. LDL, low-density lipoprotein; HDL, high-density lipoprotein; BMI, body mass index; WHR, waist to hip ratio; Hs-CRP, high sensitivity C-reactive protein; HCY, homocysteine; ECAS, extracranial atherosclerosis; aICAS, asymptomatic intracranial atherosclerosis*.

* *represent for p-value <0.05*.

## Discussion

In this study, we found that tubal ligation might be associated with a higher prevalence of aICAS in Chinese female participants over 60 years old independent of traditional cardiovascular risk factors, such as hypertension, diabetes, and dyslipidemia. To the best of our knowledge, this was the first study indicating that tubal ligation might be a potential risk factor for aICAS.

Tubal ligation was one of the most commonly used methods of sterilization which blocked ovulation physically (19). It was easy to operate and had been considered highly safe. There had been few reports on the side effects of tubal ligation before. It was estimated that a total of 180 million women of reproductive age had undergone tubal ligation globally, of which ~75% were from China and India (20). Therefore, tubal ligation was an important influencing factor with a huge potential impact due to a large number of ligatures. The tubal ligation rate was about 4.8% in this study, slightly lower than the global average, which might be due to the geographical difference of these studies.

Previous studies had observed a lack of estrogen after tubal ligation (21–23). During the operation, it would inevitably block local arterial branches, increase local blood pressure, damage the microvascular supply of the ovary, and reduce estrogen (21, 24). The effect of estrogen on atherosclerosis has been extensively studied in the context of coronary heart disease. Basic research has already delved into the selective pathways mediated by estrogen receptors and their specific roles in atherosclerosis (25). Endogenous estrogen was an important regulator for lipid metabolism and plaque progression. Several epidemiological studies had documented an inverse association between estrogen level and the incidence of cardiovascular disease (26, 27). Likewise, there was also a link between estrogen and cerebral atherosclerosis. Endogenous estrogen levels were closely related to carotid plaque vulnerability and stroke risk (28). A study based on monkey models of cerebral atherosclerosis also revealed the inhibitory effect of estrogen complexes on the development of common carotid plaques (29). These may provide a possible explanation for the correlation between tubal ligation and aICAS based on estrogen deficiency. Tubal ligation typically precedes menopause by decades, which means women with tubal ligation may have been exposed to potentially lower estrogen levels decades before menopause. Studies had shown that the significant decline in ovarian function mainly occurred in the perimenopausal period after tubal ligation, and unfavorable blood lipid status may be the result of the long-term decline in ovarian function (30). Correspondingly, the association between tubal ligation and aICAS was only significant among female participants over 60 years of age in this study. Although we did not detect estrogen levels in this study, we did observe abnormal lipid metabolism with higher LDL levels and lower HDL levels in female participants with tubal ligation, which could support this theory from the side.

Due to the lack of well-recognized animal models of ICAS, most ICAS studies were still population-based. The APAC study also tried looking into the relationship between estrogen deficient and aICAS with no association being found between new-onset aICAS and postmenopausal time (8). This result did not seem to support the hypothesis that estrogen deficiency contributed to the high prevalence of aICAS after tubal ligation. However, the APAC study concentrated on newly discovered aICAS of varying degrees of stenosis over a 2-year period, in which many aICAS were likely to be mild stricture. On the contrary, our study focused on the prevalence of aICAS with moderate to severe stenosis. It is well known that the progress of ICAS would be relatively slow over decades, which suggested that moderate-to-severe aICAS might better reflect the cumulative effects of long-term estrogen deficiency. Indeed, tubal ligation also lost its independent association with aICAS when we analyzed the mild stenosis population in our study (data not shown). At the same time, we also did not find any correlation between postmenopausal time and aICAS.

However, this explanation was still full of controversy. For unknown reasons, many studies on estrogen and atherosclerosis and estrogen replacement therapy have shown negative results (31, 32). The debate has been going on for decades, and there was still no unified conclusion. Several recent studies failed to find the impact of tubal ligation on ovarian blood supply and ovarian reserve (33–35). No hormonal changes could be observed during the perimenopausal period in some cases (36). The underlying mechanism was rarely studied. Further pieces of research are needed for a better understanding.

Notably, the prevalence of hypertension and dyslipidemia in this study was as high as 58.7 and 45.4%, respectively. Both hypertension and higher levels of LDL were well-established risk factors for aICAS. Women with tubal ligation also had a higher rate of hypertension and higher levels of LDL in this study. Although we had taken these factors into account in the logistic regression model, we should still be cautious about the scalability of the conclusion before obtaining more sufficient evidence.

Women with tubal ligation tended to have more pregnancies and childbirths. It was easy to understand that women with more pregnancies or childbirths were more likely to choose tubal ligation for contraception. Pregnancies and childbirths could have long-lasting effects on hemodynamics and lead to vascular remodeling (37). There was a positive correlation between parity and arterial intima thickness since atherosclerosis can be promoted by pregnancy-associated plasma protein A and other proinflammatory factors secreted during pregnancy (38, 39). However, neither association was found between parity and aICAS in this study nor did they have any significant influence on the correlation between tubal ligation and aICAS.

Some drugs may also have a confounding effect on this result. Oral contraceptives could increase blood lipid levels, reduce glucose tolerance, increase blood pressure, and thereby increase the risk of cardiovascular disease (40). In contrast, statins could lower blood lipid levels and reduce the risk of cardiovascular events (41). Medication-induced menopause or hormone replacement therapy also affected estrogen to a large extent with a possible impact on atherosclerosis. However, only 16 participants in this study had used lipid-lowering drugs, five participants had a history of drug-induced menopause or hormone replacement therapy, and six participants had a history of oral contraceptives. The relevant records were too few to be statistically valid, and they were not included as confounders in this analysis.

Besides, there were 43% of the participants in this study used intrauterine devices for contraception. It was thought that an intrauterine device could cause inflammation of the fallopian tubes, and the pain of intrauterine device insertion may trigger a vasovagal response (42). No previous studies had reported a correlation between intrauterine devices and atherosclerosis. In this study, we also did not observe any association between intrauterine devices and aICAS.

Intracranial atherosclerosis was different from carotid and coronary atherosclerosis in the development process, pathophysiological mechanism, and risk factors. In this study, tubal ligation was only significantly related to aICAS, not to ECAS. Menopause was associated with an increased risk of carotid atherosclerosis and cardiovascular disease (43, 44). While no significant association between menopause and aICAS was found after multivariate adjustment. In previous studies, several other MRH factors had also been reported to be associated with atherosclerosis. Early menarche and adverse pregnancy outcomes were associated with a higher risk of atherosclerotic cardiovascular disease (45, 46). Early age at first childbirth was also believed to be related to cardiovascular disease in a systematic review (47). Breastfeeding may also have a beneficial effect on subclinical atherosclerosis (48). In this study, we also analyzed the association between these factors and aICAS. None of these factors were found to be significantly associated with aICAS.

As far as we know, this was currently the most comprehensive study exploring the association between MRH and aICAS, including most of the previously reported MRH factors related to atherosclerosis. Only tubal ligation may be independently associated with aICAS for women over 60 years old in rural China. As we all know, the prevalence of ICAS varied greatly by region, both China and Africa were developing countries with a high incidence of ICAS (2). However, the proportion of traditional cardiovascular risk factors such as hypertension, diabetes, and dyslipidemia in China was not significantly higher than that of the Caucasians (2). The excess burden of ICAS remained unaccounted for. Coincidentally, three-quarters of ligated women in the world came from China and Africa (19). This was a very interesting phenomenon that provided a possible explanation for the regional epidemic differences of aICAS from a new perspective.

There were still some limitations in this study. First, this was a cross-sectional study from a single center. Only the association between tubal ligation and aICAS can be obtained, and there may be regional bias in the conclusion. Follow-up studies on large populations from multiple centers were needed to further clarify the correlation between tubal ligation and aICAS. Second, MRH was collected retrospectively through the case report form. There may be retrospective biases in data collection. Third, MRH belonged to personal privacy, and data collection required informed consent, which means inevitable selection bias.

In conclusion, the close association between tubal ligation and aICAS provides a new perspective for us to study the epidemiological characteristics of ICAS. This is a novel and noteworthy result which deserves further study.

## Data Availability Statement

The raw data supporting the conclusions of this article will be made available by the authors, without undue reservation.

## Ethics Statement

The studies involving human participants were reviewed and approved by Human Experiment Ethics Standards Committee of Shandong Provincial Hospital Affiliated to Shandong First Medical University. The patients/participants provided their written informed consent to participate in this study.

## Author Contributions

XM and SSa: Data curation and analysis and writing original draft. YZ and XJ: Data acquisition. XW and SSh: Data analysis. GW: Supervision and data curation and analysis. FX: Methodology, supervision, and conceptualization. YD: Methodology and conceptualization. ML: Methodology, conceptualization, supervision, and project administration. QS: Conceptualization, methodology, writing review and editing, supervision, and project administration. All authors contributed to the article and approved the submitted version.

## Funding

This study was supported by the grants from Jinan Science and Technology Bureau (201704101), Department of Science and Technology of Shandong Province (2016GSF201062, ZR2017MH114, and ZR2020QH109), the National Natural Science Foundation of China (8171101298 and 81971128), and the Ministry of Science and Technology of China (2017YFC1310100 and 2017YFC0907003).

## Conflict of Interest

The authors declare that the research was conducted in the absence of any commercial or financial relationships that could be construed as a potential conflict of interest.

## Publisher's Note

All claims expressed in this article are solely those of the authors and do not necessarily represent those of their affiliated organizations, or those of the publisher, the editors and the reviewers. Any product that may be evaluated in this article, or claim that may be made by its manufacturer, is not guaranteed or endorsed by the publisher.
